# The Safety and Efficacy of Tranexamic Acid in Oncology Patients Undergoing Endoprosthetic Reconstruction and a ROTEM-Based Evaluation of Their Hemostatic Profile: A Pilot Study

**DOI:** 10.3390/cancers13163951

**Published:** 2021-08-05

**Authors:** Andreas G. Tsantes, Ioannis G. Trikoupis, Dimitrios V. Papadopoulos, Stavros Goumenos, Daniele Piovani, Georgios K. Nikolopoulos, Argyri Gialeraki, Stefanos Bonovas, Panayiotis J. Papagelopoulos, Vasilios A. Kontogeorgakos, Argirios E. Tsantes

**Affiliations:** 1 Laboratory of Haematology and Blood Bank Unit, “Attiko” Hospital, School of Medicine, National and Kapodistrian University of Athens, 12462 Athens, Greece; agialer@med.uoi.gr (A.G.); atsantes@med.uoa.gr (A.E.T.); 2 First Department of Orthopaedics, School of Medicine, National and Kapodistrian University of Athens, 12462 Athens, Greece; itrikoupis@med.uoa.gr (I.G.T.); stgoumenos@med.uoa.gr (S.G.); pjportho@med.uoa.gr (P.J.P.); 3 Department of Orthopaedic Surgery, University of Pittsburgh, Orthopedic Specialists-UPMC, Pittsburgh, PA 15237, USA; papadopoulosd@upmc.edu; 4 Department of Biomedical Sciences, Humanitas University, Pieve Emanuele, 20090 Milan, Italy; daniele.piovani@humanitasresearch.it (D.P.); stefanos.bonovas@hunimed.eu (S.B.); 5 IRCCS Humanitas Research Hospital, Rozzano, 20089 Milan, Italy; 6 Medical School, University of Cyprus, Nicosia 1678, Cyprus; nikolopoulos.georgios@ucy.ac.cy

**Keywords:** endoprosthetic reconstruction, musculoskeletal oncology, tranexamic acid, rotational thromboelastometry, blood loss, hemostasis

## Abstract

**Simple Summary:**

Tranexamic acid can be an effective and safe way to reduce perioperative bleeding following an endoprosthetic reconstruction of a lower limb after a bone tumor resection. Tranexamic acid does not result in a complete shutdown of the fibrinolysis, supporting its safe use without increasing the risk of thromboembolic complications.

**Abstract:**

Background: An endoprosthetic reconstruction in musculoskeletal oncology patients is associated with significant blood loss. The purpose of this study is to evaluate the safety and efficacy of tranexamic acid (TXA) for these patients and to assess any changes in their hemostatic profile using rotational thromboelastometry (ROTEM). Methods: A retrospective observational study was performed including 61 patients with primary or metastatic bone tumors who underwent surgery. Group A (*n* = 30) received both intravenous and local TXA whereas Group B (*n* = 31) was the control group. The primary outcomes were perioperative blood loss and blood unit transfusions and the secondary outcomes included the incidence of thromboembolic complications and a change in blood coagulability as reflected by ROTEM parameters. Results: The median difference in blood loss between the two groups was 548.5 mL, indicating a 29.2% reduction in the 72 h blood loss following TXA administration (*p* < 0.001). TXA also led to a reduced transfusion of 1 red blood cell (RBC) unit per patient (*p* < 0.001). The two groups had similar rates of thromboembolic complications (*p* = 0.99). The antifibrinolytic properties of TXA were confirmed by the significantly higher INTEM, FIBTEM and EXTEM LI60 (*p* < 0.001, *p* = 0.005 and *p* < 0.001, respectively) values in the TXA group. Conclusion: Tranexamic acid was associated with a significant reduction in perioperative blood loss and transfusion requirements without a complete shutdown of the fibrinolysis. Larger studies are warranted to assess the frequency of these outcomes in musculoskeletal oncology patients.

## 1. Introduction

Extensive surgical resections and reconstructions for primary or metastatic bone tumors are associated with considerable blood loss and increased requirements for a blood transfusion [1,2,3]. Blood loss is associated with a high mortality risk, a high reoperation rate, an increased overall length of stay and increased hospital costs especially among anemic cancer patients [4]. Moreover, a blood transfusion is not without risks as it is related to several adverse events such as infection and a hemolytic transfusion reaction; therefore, several measures have been implemented in the orthopedic surgical setting to reduce perioperative blood loss [2,5].

Tranexamic acid (TXA) is a synthetic analog of lysine that prevents fibrinolysis by blocking the lysine binding sites on plasminogen and it has been extensively used over the past decade in orthopedic surgeries to decrease perioperative blood loss and the transfusion rate [6,7,8,9,10,11,12,13]. Moreover, TXA has been shown to decrease the inflammatory response resulting in less postoperative pain, less postoperative swelling and higher patient satisfaction scores [14,15]. TXA can also decrease the overall healthcare cost due to shorter hospital stays and fewer transfusions [15,16]. Although there is a long-standing concern about TXA-induced hypercoagulability that may increase the risk for postoperative venous thromboembolism (VTE), there are multiple studies even in high-risk patients supporting its safe use [5,16,17,18,19,20].

Rotational thromboelastometry (ROTEM) is a laboratory viscoelastic method that assesses clot dynamics from the clot formation to the clot breakdown and fibrinolysis. There are two main differences between conventional coagulation tests such as prothrombin time (PT) and viscoelastic tests: viscoelastic tests are performed on whole blood specimens whereas conventional tests are performed on plasma specimens. Second viscoelastic tests provide a dynamic analysis from the clot formation to the clot lysis whereas conventional tests evaluate only the initial steps of the coagulation mechanism until thrombin generation. Therefore, a ROTEM analysis could be used to monitor changes in blood coagulability such as hypofibrinolysis in those patients who receive TXA [21]. Several different ROTEM tests have been developed, focusing on different components of coagulation. An EXTEM assay evaluates the extrinsic pathway of coagulation using thromboplastin whereas an INTEM assay evaluates the intrinsic pathway using a contact activator. A FIBTEM assay assesses the fibrinogen activity through the activation of the extrinsic pathway but in the presence of a platelet function inhibitor.

The purpose of this study is to evaluate the impact of TXA on perioperative blood loss, transfusion requirements and the venous thromboembolism rate following a tumor resection and an endoprosthetic reconstruction in musculoskeletal oncology patients. Moreover, it will evaluate the pattern of hemostatic alternations in these patients using a ROTEM analysis.

## 2. Materials and Methods

### 2.1. Study Design

This was a retrospective observational study performed at the Department of Orthopedic Surgery at Attiko General University Hospital from June 2019–June 2020. The inclusion criteria were patients with primary or metastatic bone tumors who underwent a resection and an endoprosthetic reconstruction of the proximal femur, distal femur and proximal tibia. Patients with congenital or acquired coagulopathy, severe renal or liver insufficiency, previous thromboembolic complications and who received perioperative plasma products were excluded. The study was approved by the Institutional Review Board of the hospital (Ref. Number: 2020/535). Patients were divided into two groups, with or without the administration of TXA, based on the surgeon’s preference. All procedures were performed by two surgeons (V.A.K. and P.J.P.). The control group patients did not receive TXA. For the patients in the TXA group, no tourniquet control was applied and a bolus intravenous dose of TXA (1.5 g per 90 kg patient) was administered after the induction of the anesthesia. A second dose (1.5 g per 90 kg patient) was locally delivered, after skin closure, through the draining tubes. The draining tubes were connected to negative pressure bottles. The draining tubes remained closed for 2 h after the local administration of TXA. The draining tubes were removed between the third and fifth postop day in all patients of both study groups. The method of TXA application was consistent in all patients in the TXA group.

Postoperatively, all patients received low molecular weight heparin (LMWH) for thromboprophylaxis as many of these patients had received preop chemotherapy. A red blood cell (RBC) transfusion was indicated for hemoglobin levels <8 gr/dL or if there were signs of hemodynamic instability/anemia. The collected data included demographics, tumor characteristics, surgical parameters such as resection length and laboratory results. All patients had a minimum of 2-week, 6-week and 12-week follow-up appointments. The primary outcome of the study was the efficacy of TXA as evaluated by a reduction in perioperative blood loss. Secondary outcomes included the safety of TXA based on the incidence of thromboembolic complications and changes in the coagulation profile of these patients due to TXA as reflected by the ROTEM parameters.

### 2.2. Efficacy and Safety

Perioperative blood loss was determined based on: (i) a drop in hemoglobin levels in the first 72 h, (ii) the number of transfused red blood cell (RBC) units and (iii) 72 h total blood loss as calculated by the Hemoglobin Balance method, which is a reliable and accurate method of calculating bleeding taking into account various parameters [22]. Regarding safety, patients were monitored for the development of VTE events (pulmonary embolism and deep vein thrombosis) for 3 months following surgery. Pulmonary embolisms and deep vein thromboses were diagnosed with a CT angiography and venous Triplex, respectively, in symptomatic patients.

### 2.3. Hemostatic Profile

Conventional laboratory assays such as a platelet (PLT) count, prothrombin time (PT) and activated partial thromboplastin time (APTT) were recorded. A ROTEM analysis was also performed for these high-risk procedures as part of an investigation protocol for the coagulation profile of high-risk patients in our hospital. Blood samples were collected for each patient at two perioperative times: preoperatively and postoperatively on the day of surgery. For the ROTEM analysis, a citrated whole blood sample was analyzed in a ROTEM analyzer (ROTEM delta, Tem Innovations GmbH, Munich, Germany) within 90 min of the blood draw as formerly described [23]. The ROTEM analysis included EXTEM, INTEM and FIBTEM assays. The following ROTEM parameters were measured: clotting time (CT, seconds), the time from the start of the analysis until a clot formation of 2 mm in amplitude was reached, clot formation time (CFT, seconds), the time from CT (amplitude of 2 mm) until a clot firmness of 20 mm was reached, amplitude (recorded at 10 min (A10, mm)), maximum clot firmness (MCF, mm), the percentage of lysis representing the maximum fibrinolysis and the lysis index at 60 min (LI60, %), which is the percentage of the remaining clot stability in relation to the MCF following the 60 min observation period after CT that indicates the speed of fibrinolysis.

### 2.4. Statistical Analysis

The statistical analysis included descriptive statistics of patients for baseline demographics, surgical parameters, parameters regarding perioperative blood loss and laboratory results. Data were presented as means ± standard deviations (SD), medians and interquartile ranges (IQR) or percentages when appropriate. These variables were compared between the two study groups using the non-parametric Wilcoxon rank sum test and the chi-squared test when appropriate. Moreover, the correlation between the ROTEM parameters and perioperative blood loss was evaluated by the non-parametric Spearman’s rank test. A Spearman’s rho of <0.20 indicated a very weak correlation, 0.21 to 0.40 a weak correlation, 0.41 to 0.60 a moderate correlation, 0.61 to 0.80 a strong correlation and >0.81 a very strong correlation. A multivariable linear regression analysis adjusted for age, gender, body mass index (BMI), resection type (proximal femur vs. distal femur vs. proximal tibia) and resection length was performed in order to assess whether the administration of TXA was significantly associated with changes in perioperative blood loss, the number of transfusions per patient and hypofibrinolysis as evaluated by the ROTEM values. STATA version 15.0 (Stata Corp., College Station, TX, USA) software was used for the statistical analysis. For all tests, a *p*-value lower than 0.05 indicated a statistical significance.

## 3. Results

A total of 71 patients were initially reviewed. Four patients did not meet the inclusion criteria and were excluded from the study because of previous VTE events or coagulopathy. A total of 67 were included, 34 in the control group and 33 in the TXA group. One patient who was lost to follow-up and two patients who received fresh frozen plasma were excluded from the control group. One patient who died in the TXA group and two patients who received fresh frozen plasma were also excluded from the TXA group. The final analyzed cohort of patients consisted of 61 patients comprising 30 patients in the TXA group and 31 patients in the control group. The median age of patients in the TXA group was 60 (interquartile range (IQR), 39–67) and the median age of patients in the control group was 39 (IQR, 28–56; *p* = 0.058). The most common diagnosis was a metastatic carcinoma (33.3%) in the TXA group whereas it was an osteosarcoma in the control group (48.3%)**.** The tumor was located in the proximal femur in 12 (40%) patients of the TXA group vs. 11 (35.4%) patients of the control group, in the distal femur in 15 (50%) patients of the TXA group vs. 17 patients of the control group and in the proximal tibia in 3 (10%) patients of the TXA group vs. 3 (9.6%) patients of the control group. The two groups did not significantly differ regarding the preoperative diagnosis or the location of the tumor (*p* > 0.05; Table 1). The median resection length in the TXA group was 16 cm (IQR, 15–18 cm) and in the control group it was 17.0 cm (IQR, 15–18 cm; *p* = 0.81). The demographics and clinical parameters of the study population are summarized in Table S1 and Table 1. The median preoperative Hb was 11.2 g/dL (IQR, 10.5–12.2 g/dL) in the TXA group and 11.7 g/dL (IQR, 10.8–12.0 g/dL) in the control group (*p* = 0.79). The median preoperative PLT counts in the TXA group and the control group were 266.0 × 103/mL (IQR, 229–326 × 103/mL) and 284.5 × 103/mL (IQR, 212–357 × 103/mL), respectively (*p* = 0.62) and the median preoperative PT was 11.5 s (IQR, 10.8–13.2 s) in the TXA group and 11.8 s (IQR, 11.1–12.6 s) in the control group (*p* = 0.83). The conventional preoperative laboratory values are presented in the Supplementary Table.

The median blood loss in the first 72 h for the TXA group was 1324.5 mL (IQR, 1104–1511 mL) vs. 1873 mL (IQR, 1711–2153 mL) in the control group (*p* < 0.001). Therefore, the median difference in blood loss between the two groups was 548.5 mL, indicating a 29.2% reduction in the 72 h blood loss following TXA administration. The median number of transfused RBC units was 2 (IQR, 1–2) per patient in the TXA group and 3 (IQR, 2–3) per patient in the control group (*p* < 0.001), showing a median reduction of 1 RBC unit per patient with TXA use. Moreover, 24 patients in the TXA group (80.0%) required transfusions compared with all 31 patients in the control group (100.0%), resulting in a 20.0% reduction in the transfusion incidence with TXA administration (*p* = 0.009). The postoperative drop in Hb concentration was higher (*p* < 0.001) in the control group (median: 3.8 g/dL; IQR, 2.7–4.5 g/dL) compared with the TXA group (median: 2.4 g/dL; IQR, 2.1–2.9 g/dL). Table 2 presents the parameters of perioperative blood loss in both groups. Moreover, the multivariable linear regression analysis (adjusted for gender, age, BMI, type of resection and resection length) further confirmed that the use of TXA resulted in significantly lower perioperative blood loss (*p* < 0.001), a lower number of transfusions per patient (*p* = 0.002) and a lower postoperative drop in Hb concentration (*p* = 0.013; Table 3). There was one patient in the control group with a pulmonary embolism during the follow-up and there were no thromboembolic complications in the TXA group (*p* = 0.99).

The preoperative ROTEM parameters were similar between the two groups, revealing a similar baseline coagulation profile. Most of the postoperative ROTEM parameters were also similar (Table 4).

**Figure 1:**
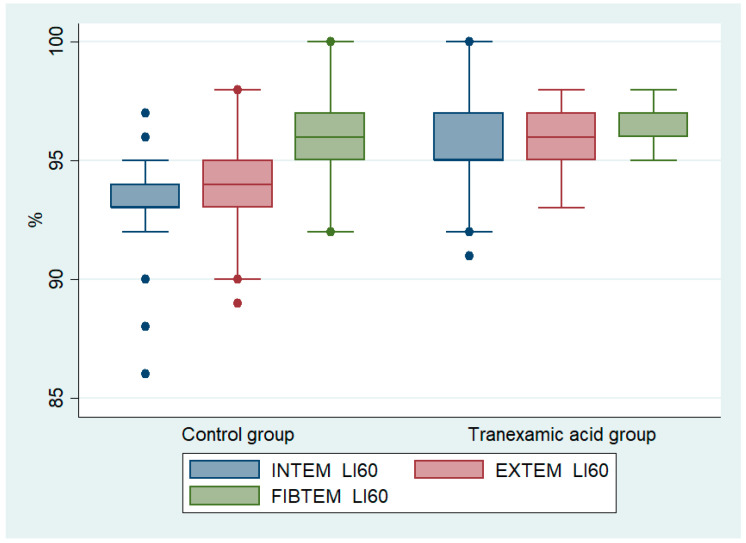
Boxplots of INTEM, EXTEM and FIBTEM LI60 results of patients with and without the use of tranexamic acid.

Certain postoperative ROTEM parameters significantly differed, indicating a lower fibrinolysis activity for those patients who received TXA (Table 5). Specifically, LI60 was significantly higher in the TXA group compared with the control group for the INTEM (medians: 95% vs. 93%, *p* < 0.001), EXTEM (medians: 96% vs. 94%, *p* < 0.001) and FIBTEM (medians: 97% vs. 96%, *p* = 0.005) assays (Figure 1). However, TXA use did not result in a fibrinolysis shutdown, which has been defined as EXTEM LI60 values ≥98% [24,25,26,27,28].

The multivariable linear regression analysis (adjusted for gender, age, BMI, type of resection and resection length; Table 6) further confirmed that TXA administration resulted in a significantly higher INTEM LI60 (*p* = 0.001), EXTEM LI60 (*p* < 0.001) and FIBTEM LI60 (*p* = 0.010). Perioperative blood loss was weakly correlated with lower INTEM LI60 (rho = −0.38, *p* = 0.002) and lower FIBTEM LI60 (rho = −0.36, *p* = 0.003) values and moderately correlated with a lower EXTEM LI60 (rho = −0.41, *p* = 0.001).

## 4. Discussion

The application of TXA in the orthopedic surgical setting has gained ground over the past years. This is the first study to evaluate the intravenous and local use of TXA following a tumor resection and an endoprosthetic reconstruction and it is also the first study to use a viscoelastic assay to assess the hemostatic profile of these patients in order to provide a more detailed insight on the effect of TXA on the coagulation mechanism. Our study showed that TXA significantly reduced perioperative blood loss by 29.2% in the first 72 h and also resulted in a 20% reduction in the transfusion rate. The antifibrinolytic effect of TXA that resulted in blood loss reduction was depicted by the results of the ROTEM analysis according to which the significantly higher LI60 values in TXA patients indicated a marked reduction of the clot breakdown. Additionally, TXA did not result in a higher rate of thromboembolic complications. A possible explanation for this can be also given by the results of the ROTEM analysis. Although a lower fibrinolytic activity was evident in TXA patients, these patients did not develop a fibrinolysis shutdown (based on the ROTEM-determined definition), which has been associated with an increased risk for a deep vein thrombosis in orthopedic surgery [29]. Moreover, patients who received TXA were not in a higher prothrombotic state compared with the control group, as shown by the similar A10 and MCF values between the two groups, which may further explain why TXA was not associated with increased VTE events.

Although ample research has been conducted regarding the safety and efficacy of perioperative TXA in patients undergoing a hip or knee arthroplasty, there is a lack of evidence regarding its use in certain high-risk patients such as oncology patients. Nonetheless, despite the lack of literature to support its use in this cohort of patients, several surgeons within the musculoskeletal oncology community use TXA for these high blood loss procedures [1]. Whiting et al., in a cohort of 402 high-risk patients for thromboembolic complications who underwent total joint replacements, found that the intravenous administration of TXA did not result in a significantly higher VTE rate [19]. In the largest study so far, including high-risk patients for a postoperative venous thromboembolism, the safety of TXA was evaluated in 8877 patients who underwent a hip or knee arthroplasty [18]. The authors of this study also found that high-risk patients who received TXA had no statistically significant difference in the odds of developing VTE events compared with those who did not receive TXA. Haase et al. investigated TXA use in 90 cancer patients who underwent bone tumor resections and endoprosthetic reconstructions. However, there was no assessment of the coagulation profile in the patients included in this study [1]. Patients who received TXA experienced a 36% reduction in the 72 h calculated mean blood loss; the average blood transfusions decreased by 0.45 RBC units per patient in the TXA group and the transfusion incidence decreased by 21.1%. It is noteworthy, however, that despite the similar rates of blood loss reduction, our patients received both local and intravenous TXA whereas patients in that study received only local intraarticular TXA. The similar rates of blood loss reduction despite the different routes of TXA administration between the two studies may be attributed to the fact that although Haase et al. conducted a larger study with more patients, their results were not adjusted for confounding factors. Therefore, larger randomized controlled studies are needed to evaluate the impact of the intravenous use of TXA on blood loss reduction in these clinical settings.

The application of viscoelastic methods to identify and monitor changes in the hemostatic profile of patients receiving TXA has been evaluated in only a few studies and never before in musculoskeletal oncology patients. Wu et al. evaluated the results of TXA using thromboelastography (TEG) in 359 patients who underwent a total hip or a total knee arthroplasty by comparing a multiple dose and a single dose of TXA [30]. Patients with multiple doses had a significantly shorter R time and a TEG parameter similar to the CT in the ROTEM method whereas all other TEG parameters and conventional coagulation parameters were similar. The incidence of VTE events was also similar for the two groups. The authors recommended multiple doses in clinical practice as they stated that while multiple doses are related to an aggravated hypercoagulable state compared with a single dose, they did not provoke thromboembolic complications when an appropriate thromboprophylaxis was used. In another study, Xu et al. also used TEG to evaluate the dynamic changes in blood coagulation of patients who underwent THA following TXA administration [31]. The authors enrolled 207 patients and compared no use with the local and intravenous administration of TXA. The local TXA administration did not affect the TEG parameters whereas the intravenous TXA administration significantly affected the TEG parameters, resulting in lower R and K times and in a higher maximum amplitude (MA) and angle values indicating a hypercoagulable state. However, the rates of the VTE events were similar among the three groups. Although the authors of this study found that intravenous TXA promoted clot formation (a significant decrease in R and K times) and increased clot strength (a significant increase in the α angle and MA), they did not mention any effect on the fibrinolytic parameters although fibrinolysis is the main component of hemostasis that TXA affects. As opposed to the results of this study, we showed that although TXA affected fibrinolysis, clot formation and clot strength were not affected. Our results are in line with the literature regarding the clinical safety of TXA use as the similar risk for thromboembolic complications with or without TXA may be related to the fact that TXA does not result in a prothrombotic state.

There are a few limitations of our study that are worth mentioning. First, the number of participants in our study was relatively small; therefore, larger studies are needed to reach definite conclusions regarding the efficacy and safety of TXA in musculoskeletal oncology patients. However, due to the lack of relevant data in the literature, the results of our study are valuable to surgeons within the musculoskeletal oncology community. Second, this was a retrospective study and our patients were not randomly assigned into the two groups. This has the risk of heterogeneity between the two study groups and certain covariates such as resection length or BMI can confound the relationship between TXA use and blood loss or between TXA use and the ROTEM parameters. However, the two groups had similar preoperative demographics and laboratory values and, in addition, we performed a regression analysis to adjust the evaluation of these relationships for several covariates. Last, the measurement of TXA levels (or its metabolites) in blood and an evaluation of the relationship between different TXA blood levels and the coagulation/hematological profile of the patients was not performed. This would be valuable as it would allow us to define the dose of TXA that results in an ideal risk–benefit equilibrium.

## 5. Conclusions

In conclusion, the results of this pilot study indicate that the use of TXA in bone tumor resections and an endoprosthetic reconstruction of proximal femur, distal femur and proximal tibia is a safe and effective measure to reduce perioperative blood loss and transfusion requirements. The antifibrinolytic mechanism of the action, which is responsible for this reduction, was further confirmed by the ROTEM parameters. Most important, the results of the ROTEM analysis showed that although TXA administration resulted in a lower fibrinolytic activity, it did not lead to fibrinolysis shutdown and also the clot firmness was not affected. The latter ROTEM results may explain why TXA did not increase the risk for VTE events, supporting its safe use.

## Figures and Tables

**Table 1:** Tumor characteristics and surgical parameters of the study population.

Parametters	Total (*n* = 61)	TXA Group (*n* = 30)	Control Group (*n* = 31)	*p*-Value
Primary diagnosis				
Osteosarcoma	24 (39.3)	9 (30)	15 (48.3)	0.14
Metastatic carcinoma	18 (29.5)	10 (33.3)	8 (25.8)	0.57
Multiple myeloma	4 (6.5)	3 (10)	1 (3.2)	0.28
Chondrosarcoma	13 (21.3)	8 (26.7)	5 (16.7)	0.31
Type of resection				
Proximal femur	23 (37.7)	12 (40.0)	11 (35.4)	0.71
Distal femur	32 (52.4)	15 (50.0)	17 (54.8)	0.7
Proximal tibia	6 (9.8)	3 (10.0)	3 (9.6)	0.96
Resection length (cm)	16.5 (15.0–18.0)	16.0 (15.0–18.0)	17.0 (15.0–18.0)	0.81

Data are presented as medians and interquartile ranges (IQR) or as absolute values (percentages) when appropriate. The non-parametric Wilcoxon rank sum test and the chi-squared test were used for the comparison between the two groups.

**Table 2:** Blood loss parameters for the two study groups.

Variables	TXA Group (*n* = 30)	Control Group (*n* = 31)	*p*-Value
Total blood loss (mL)	1324.5 (1104.0–1511.0)	1873.0 (1711.0–2153.0)	<0.001
PFR	1404.5 (1292.5–1565.5)	2112.0 (1898.0–2523.0)	<0.001
DFR	1242.0 (1036.0–1486.0)	1834.0 (1703.0–1945.0)	<0.001
PTR	1209.0 (1167.0–1321.0)	1436.0 (718.0–1901.0)	0.51
Average number of transfusions	2.0 (1.0–2.0)	3 (2.0–3.0)	<0.001
Transfusion incidence	24 (80)	31 (100)	0.009
Hb drop (g/dL)	2.4 (2.1–2.9)	3.8 (2.7–4.5)	<0.001

Abbreviations: PFR, proximal femoral replacement; DFR, distal femoral replacement; PTR, proximal tibial replacement; RBC, red blood cell; Hb, hemoglobin. Data are presented as medians and interquartile ranges (IQR). The non-parametric Wilcoxon rank sum test was used for the comparison between the two groups.

**Table 3:** Results of a multivariable regression analysis for the evaluation of the effect of TXA on blood loss parameters adjusted for age, gender, BMI, resection length and type of resection (proximal femur, distal femur, proximal tibia).

Variables	Use of TXA		
	Coefficient	95% CI	*p*-Value
Perioperative blood loss (mL)	−717.5	−956.6–−478.5	<0.001
RBC units transfused per patient	−1.03	−1.64–−0.41	0.002
Hb drop (g/dL)	−1.01	−1.78–−0.23	0.013

Abbreviations: RBC, red blood cell; Hb, hemoglobin; CI, confidence interval.

**Table 4:** Preoperative and postoperative ROTEM parameters of the two groups.

ROTEM Parameters	Preoperative			Postoperative		
	Control Group (*n* = 31)	TXA Group (*n* = 30)	*p*-Value	Control Group (*n* = 31)	TXA Group (*n* = 30)	*p*-Value
INTEM CT (s)	179 (177–180)	177 (175.0–180.0)	0.07	175 (173.0–177.0)	173.5 (170.0–176.0)	0.13
INTEM CFT (s)	65.0 (63.0–70.0)	65.5 (64.0–67.0)	0.74	60 (57.0–63.0)	62 (61.0–63.0)	0.07
INTEM MCF (mm)	72 (69.0–75.0)	73 (72.0–75.0)	0.09	75 (73.0–76.0)	75 (73.0–77.0)	0.70
INTEM A10 (mm)	68.0 (66.0–70.0)	68 (66.0–70.0)	0.68	70.0 (69.0–72.0)	69.0 (68.0–70.0)	0.07
INTEM LI60 (%)	94 (92.0–96.0)	94 (93.0–96.0)	0.61	93 (93.0–94.0)	95 (95.0–97.0)	<0.001
EXTEM CT (s)	64 (62.0–67.0)	65 (62.0–68.0)	0.19	63 (61.0–65.0)	64 (63.0–65.0)	0.62
EXTEM CFT (s)	50.0 (47.0–53.0)	51.0 (48.0–53.0)	0.83	49.0 (47.0–51.0)	50.0 (49.0–52.0)	0.12
EXTEM MCF (mm)	71.0 (67.0–74.0)	70.5 (68.0–74.0)	0.98	73.0 (71.0–74.0)	72 (71.0–73.0)	0.55
EXTEM A10 (mm)	63.0 (61.0–65.0)	65.0 (62.0–66.0)	0.08	65.0 (63.0–67.0)	68.0 (62.0–70.0)	0.10
EXTEM LI60 (%)	92.0 (92.0–94.0)	93 (92.0–95.0)	0.08	94.0 (93.0–95.0)	96.0 (95.0–97.0)	<0.001
FIBTEM CT (s)	59.5 (57.5–62.5)	58.0 (55.5–60.5)	0.29	58.0 (57.0–59.0)	57.0 (56.0–58.0)	0.27
FIBTEM MCF (mm)	19 (17.0–21.0)	20 (18.0–21.0)	0.53	17.0 (16.0–19.0)	18 (16.0–20.0)	0.53
FIBTEM A10 (mm)	13.0 (10.0–15.0)	14.0 (13.0–15.0)	0.38	10.0 (8.0–11.0)	11.5 (10.0–12.0)	0.057
FIBTEM LI60 (%)	95.0 (91.5–96.5)	94.0 (94.0–95.0)	0.33	96.0 (95.0–97.0)	97.0 (96.0–97.0)	0.005

Abbreviations: CT, clotting time; CFT, clot formation time; A10, clot amplitude at 10 min; MCF, maximum clot firmness; LI60, lysis index at 60 min. Data are presented as medians and interquartile ranges (IQR). The non-parametric Wilcoxon rank sum test was used for the comparison between the two groups.

**Table 5:** Altered postoperative ROTEM parameters between the two groups.

ROTEM Parameters	Control Group (*n* = 31)	TXA Group (*n* = 30)	*p*-Value
Postoperative INTEM LI60 (%)	93 (93.0–94.0)	95 (95.0–97.0)	<0.001
Postoperative EXTEM LI60 (%)	94.0 (93.0–95.0)	96.0 (95.0–97.0)	<0.001
Postoperative FIBTEM LI60 (%)	96.0 (95.0–97.0)	97.0 (96.0–97.0)	0.005

Abbreviations: LI60, lysis index at 60 min. Data are presented as medians and interquartile ranges (IQR). The non-parametric Wilcoxon rank sum test was used for the comparison between the two groups.

**Table 6:** Results of a multivariable regression analysis for the evaluation of the effect of TXA on the ROTEM parameters adjusted for age, gender, BMI, resection length and type of resection (proximal femur, distal femur, proximal tibia).

Variables	Use of TXA		
	Coefficient	95% CI	*p*-Value
INTEM LI60 (%)	2.26	0.98–3.55	0.001
FIBTEM LI60 (%)	0.90	0.22–1.58	0.010
EXTEM LI60 (%)	1.99	1.06–2.91	<0.001

Abbreviations: LI60, lysis index at 60 min; CI, confidence interval.

## Data Availability

The data presented in this study are available on request from the corresponding author.

## Supplementary Materials

The following are available online at https://www.mdpi.com/article/10.3390/cancers13163951/s1; Table S1. Baseline characteristics of the study population.

## References

[1] Haase D., Templeton K., Rosenthal H., Sweeney K. (2020). Tranexamic acid in patients with cancer undergoing endoprosthetic reconstruction. J. Am. Acad. Orthop. Surg..

[2] Sabatini L., Atzori F., Revello S., Scotti L., Debiasi F., Massè A. (2014). Intravenous use oftranexamic acid reduces postoperative blood loss in total knee arthroplasty. Arch. Orthop. Trauma Surg..

[3] Borisov D.B., Iudin S.V., Tiuriapin A.A., Kapinos A.A., Vyl’iurov I.V., Kazakevich E.V. (2010). Prevention and treatment of anemia during endoprosthetic replacement of large joints. Anesteziol. Reanimatol..

[4] Vera-Llonch M., Hagiwara M., Oster G. (2006). Clinical and economic consequences of bleeding following major orthopedic surgery. Thromb. Res..

[5] Wei W., Wei B. (2014). Comparison of topical and intravenous tranexamic acid on blood loss and transfusion rates in total hip arthroplasty. J. Arthroplast..

[6] Aguilera X., Martinez-Zapata M.J., Bosch A., Urruita G., González J.C., Jordan M., Gich I., Maymó R.M., Martínez N., Monllau J.C. (2013). Efficacy and safety of fibrin glue and tranexamic acid to prevent postoperative blood loss in total knee arthroplasty: A randomized controlled clinical trial. J. Bone Jt. Surg. Am..

[7] Bidolegui F., Arce G., Lugones A., Pereira S., Vindver G. (2014). Tranexamic acid reduces blood loss and transfusion in patients undergoing total knee arthroplasty without Tourniquet: A prospective randomized controlled trial. Open Orthop. J..

[8] Chang C.H., Chang Y., Chen D.W., Ueng S.W.N., Lee M.S. (2014). Topical tranexamic acid reduces blood loss and transfusion rates associated with primary total hip arthroplasty. Clin. Orthop. Relat. Res..

[9] Zeng Y., Shen B., Yang J., Zhou Z., Kang P., Pei F. (2016). Tranexamic acid administration in primary total hip arthroplasty: A randomized controlled trial of intravenous combined with topical versus single-dose intravenous administration. J. Bone Jt. Surg Am..

[10] Fraval A., Effeney P., Fiddelaers L., Smith B., Towell B., Tran P. (2017). OBTAIN A: Outcome Benefits of Tranexamic Acid in Hip Arthroplasty. A Randomized Double-Blinded Controlled Trial. J. Arthroplast..

[11] Kelley T.C., Tucker K.K., Adams M.J., Dalury D.F. (2014). Use of tranexamic acid results in decreased blood loss and decreased transfusions in patients undergoing staged bilateral total knee arthroplasty. Transfusion.

[12] Fillingham Y.A., Ramkumar D.B., Jevsevar D.S., Yates J.A., Shores P., Mullen K., Bini S.A., Clarke H.D., Schemitsch E., Johnson R.L. (2018). The safety of tranexamic acid in total joint arthroplasty: A direct meta-analysis. J. Arthroplast..

[13] Samujh C., Falls T.D., Wessel R., Smith L., Malkani A.L. (2014). Decreased blood transfusion following revision total knee arthroplasty using tranexamic acid. J. Arthroplast..

[14] Goyal N., Chen D.B., Harris A.I., Rowden N., Kirsh G., MacDessi S.J. (2016). Clinical and financial benefits of intra-articular tranexamic acid in total knee arthroplasty. J. Orthop. Surg..

[15] Huang Z., Ma J., Shen B., Pei F. (2014). Combination of intravenous and topical application of tranexamic acid in primary total knee arthroplasty: A prospective randomized controlled trial. J. Arthroplast..

[16] Chimento G., Huff T., Ochsner J.L., Meyer M., Brandner L., Babin S. (2013). An evaluation of the use of topical tranexamic acid in total knee arthroplasty. J. Arthroplast..

[17] Karam J.A., Bloomfield M.R., DiIorio T.M., Irizarry A.M., Sharkey P.F. (2014). Evaluation of the efficacy and safety of tranexamic acid for reducing blood loss in bilateral total knee arthroplasty. J. Arthroplast..

[18] Porter S.B., White L.J., Osagiede O., Robards C.B., Spaulding A. (2020). Tranexamic acid administration is not associated with an increase in complications in high-risk patients undergoing primary total knee or total hip arthroplasty: A retrospective case-control study of 38,220 patients. J. Arthroplast..

[19] Whiting D.R., Gillette B.P., Duncan C., Smith H., Pagnano M.W., Sierra R.J. (2014). Preliminary results suggest tranexamic acid is safe and effective in arthroplasty patients with severe comorbidities. Clin. Orthop. Relat. Res..

[20] Drakos A., Raoulis V., Karatzios K., Doxariotis N., Kontogeorgakos V., Malizos K., Varitimidis S.E. (2016). Efficacy of local administration of tranexamic acid for blood salvage in patients undergoing intertrochanteric fracture surgery. J. Orthop. Trauma.

[21] Τsantes A.G., Trikoupis G., Papadopoulos D.V., Tsantes K.A., Mavrogenis A.F., Koulovaris P., Savvidou O.D., Kontogeorgakos V.A., Piovani D., Kriebardis A.G. (2021). Higher coagulation activity in hip fracture patients: A case-control study using rotational thromboelastometry. Int. J. Lab. Hematol..

[22] Foss N.B., Kehlet H. (2006). Hidden blood loss after surgery for hip fracture. J. Bone Jt. Surg. Br. Vol..

[23] Sokou R., Piovani D., Konstantinidi A., Tsantes A.G., Parastatidou S., Lampridou M., Ioakeimidis G., Gounaris A., Iacovidou N., Kriebardis A.G. (2020). A risk score for predicting the incidence of hemorrhage in critically ill neonates: Development and validation study. Thromb. Haemost..

[24] Lampridou M., Sokou R., Tsantes A.G., Theodoraki M., Konstantinidi A., Ioakeimidis G., Bonovas S., Politou M., Valsami S., Iliodromiti Z. (2020). ROTEM diagnostic capacity for measuring fibrinolysis in neonatal sepsis. Thromb. Res..

[25] Stettler G.R., Moore E.E., Moore H.B., Nunns G.R., Silliman C.C., Banerjee A., Sauaia A. (2019). Redefining postinjury fibrinolysis phenotypes using two viscoelastic assays. J. Trauma Acute Care Surg..

[26] Tsantes A.E., Frantzeskaki F., Tsantes A.G., Rapti E., Rizos M., Kokoris I.S., Paramythiotou E., Katsadiotis G., Karali V., Flevari A. (2020). The haemostatic profile in critically ill COVID-19 patients receiving therapeutic anticoagulant therapy. Medicine.

[27] Tsantes A.G., Papadopoulos D.V., Trikoupis I.G., Goumenos S., Piovani D., Tsantes K.A., Mavrogenis A.F., Vaiopoulos A.G., Koulovaris P., Nikolopoulos G.K. (2021). The procoagulant effect of COVID-19 disease on the thrombotic risk of patients with hip fractures due to enhanced clot strength and fibrinolysis shutdown. J. Clin. Med..

[28] Tsantes A.E., Tsantes A.G., Kokoris S.I., Bonovas S., Frantzeskaki F., Tsangaris I., Kopterides P. (2020). COVID-19 Infection-Related Coagulopathy and Viscoelastic Methods: A Paradigm for Their Clinical Utility in Critical Illness. Diagnostics.

[29] Moore H.B., Moore E.E., Gonzalez E., Chapman M.P., Chin T.L., Silliman C.C., Banerjee A., Sauaia A. (2014). Hyperfibrinolysis, physiologic fibrinolysis, and fibrinolysis shutdown: The spectrum of postinjury fibrinolysis and relevance to antifibrinolytic therapy. J. Trauma Acute Care Surg..

[30] Wu X.-D., Chen Y., Tian M., He Y., Tao Y.-Z., Xu W., Cheng Q., Chen C., Liu W., Huang W. (2019). Application of thrombelastography (TEG) for safety evaluation of tranexamic acid in primary total joint arthroplasty. J. Orthop. Surg. Res..

[31] Xu X., Jiang J., Liu W., Li X., Lu H. (2019). Application of thromboelastography to evaluate the effect of different routes administration of tranexamic acid on coagulation function in total hip arthroplasty. J. Orthop. Surg. Res..

