# Supplementary material for: The Safety and Efficacy of Tranexamic Acid in Oncology Patients Undergoing Endoprosthetic Reconstruction and a ROTEM-Based Evaluation of Their Hemostatic Profile: A Pilot Study
Source: Cancers (Basel). 2021 Aug 5;13(16):3951. doi: 10.3390/cancers13163951 (PMC8392365; doi:10.3390/cancers13163951)
Supplement: Supplementary file 1 [file cancers-13-03951-s001.zip › cancers-1324260-supplementary.pdf]

Table S1. Baseline characteristics of the study population.

	Total (n=61)	TXA group (n=30)	Control group (n=31)	p-value
Age (years)	53 (22-64.5)	60 (39-67)	39 (28-56)	0.058
Gender (males%)	34 (55.7)	17 (56.6)	17 (54.8)	0.88
BMI (Kg/m^2)	23.0 (22.0-27.0)	23.0 (21.0-27.0)	23 (22.0-25.0)	0.79
Hb (g/dl)	11.5 (10.5-12.1)	11.2 (10.5-12.2)	11.7 (10.8-12.0)	0.79
PLT (count x103/ml)	278.0 (221.0-332.0)	266.0 (229.0-326.0)	284.5(212.0-357.0)	0.62
PT (s)	11.7 (11.0-12.8)	11.5 (10.8-13.2)	11.8 (11.1-12.6)	0.83
APTT (s)	30.1 (28.3-31.5)	30.3 (27.7-32.2)	30.2 (28.4-31.2)	0.84

Abbreviations: BMI, Body Mass Index; Hb, Hemoglobin; PLTs, platelets; PT, prothrombin time; APTT, activated partial thromboplastin time
Data are presented as medians and interquartile ranges (IQR), or as absolute values (percentages) when appropriate. The nonparametric Wilcoxon rank sum test and the chi square test were used for the comparison between the 2 groups.
